# Molecular Ordering and Hydration in Model Lipid Rafts Studied by Vibrational Sum Frequency Spectroscopy

**DOI:** 10.1021/acs.jpcb.6c01062

**Published:** 2026-06-26

**Authors:** Jonas Hedberg, C. Magnus Johnson

**Affiliations:** † Division of Surface and Corrosion Science, Department of Chemistry, KTH Royal Institute of Technology, SE-100 44 Stockholm, Sweden; ‡ Surface Science Western, Western University, London, Ontario N6G 0J3, Canada

## Abstract

Vibrational sum frequency spectroscopy (VSFS) has been employed to study the molecular order, orientation, and hydration in model lipid rafts consisting of monolayers on water of deuterated DSPC (d-DSPC), cholesterol, and the sphingolipid GlcCer­(d18:1/18:0), compounds that are believed to be enriched in lipid rafts. Experiments have been carried out at surface pressures of 4, 30, and 55 mN/m for GlcCer and at 30 mN/m for cholesterol, d-DSPC, the 50/50 binary mixture of GlcCer/d-DSPC, and the ternary mixture, and the orientation of the terminating methyl group of d-DSPC has been calculated. At all surface pressures and in all mixtures, the hydrocarbon chains exhibited a well-ordered molecular structure with few gauche defects, indicating that mixtures of these constituents have the ability to form ordered structures, which lipid rafts are believed to form. In addition, the orientation of the methyl group in d-DSPC remained fairly constant in all mixtures. The water –OH stretching region (∼3000–3800 cm^–1^) was dominated by the effect of d-DSPC being the only charged molecule and hence with a large ability to orient surface water molecules.

## Graphical abstract

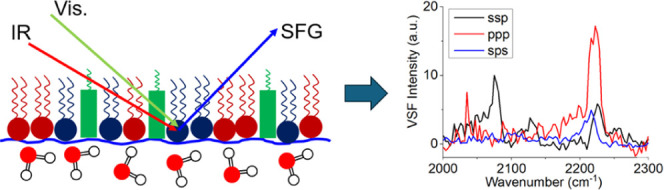

## Introduction

For a long time, cell membranes were assumed to consist of randomly distributed lipids with proteins being present as islands in the membrane.^1^ However, it has later been proposed that the cell membrane consists of disordered parts as well as highly ordered microdomains, denoted lipid rafts.^2^ These rafts are not susceptible to extraction with nonionic detergents (e.g., Triton X-100) in contrast to more disordered parts of the plasma membrane.^3^ Hence, lipid rafts are also denoted as detergent-resistant membranes (DRM). Lipid rafts are enriched in cholesterol and sphingolipids and contain phospholipids with long, saturated hydrocarbon chains.^2^ The existence of lipid rafts has been under debate for decades, although most researchers nowadays acknowledge their presence in cell membranes.^2^ Several functions of lipid rafts have been assigned, such as in the regulation of signal transduction, since the rafts contain proteins involved in signaling, and they also affect the rigidity of the membrane.^2^ Due to the complexity of natural membranes that contain a huge amount of different lipids, cholesterol, and proteins, it is difficult to study the behavior and role of individual phospholipids, for example. A way to overcome this problem is to build simple models of cell membranes, consisting of only one or a few different species. This method, in combination with the use of vibrational sum frequency spectroscopy (VSFS), allowed detailed molecular studies of interactions involving phospholipids as well as other important biological molecules.^4–30^ Most of the work has involved phospholipids belonging to the family phosphatidylcholines, but in some studies, cholesterol^17,20–23^ and sphingolipids^18,22^ have additionally been studied. By deuterating one of the species in mixtures, it is possible to examine the molecular structure of different species individually, for example, in mixtures consisting of two phospholipids.^10^

To simulate a natural lipid raft, mixtures of a long-chain phospholipid (DSPC), cholesterol, and a sphingolipid (GlcCer­(d18:1/18:0)) have been studied in this article with VSFS. The focus has been on investigating the molecular packing, orientation, and hydration of the individual components as well as mixtures thereof in order to examine how these properties are affected when going from single- to three-component systems. The most extensive studies have been performed on GlcCer since it has been the least investigated of these biomolecules.

## Theory VSFS

Vibrational sum frequency spectroscopy is a surface analytical technique, which can be applied to studies of all interfaces accessible by the laser beams involved, such as the liquid/air, solid/liquid, and solid/solid interfaces. The theory has been described in detail in several sources.^31,32^ Briefly, VSFS involves the absorption of two photons, one at a fixed visible frequency and one tunable in the infrared region, and can be viewed as a combination of an IR and a Raman process. If two centrosymmetric media are in contact with each other (e.g., air and a liquid as in the article), the sum frequency signal is under the electric dipole approximation, solely generated at the interface. This is due to the fact that the SFG (sum frequency generation) signal is zero in media with no net molecular orientation, such as in air or water, and is described in eq 1.

1 $$\chi_{R}^{\left(2\right)}=\frac{N}{\varepsilon_{0}}\left\langle \beta_{R}^{\left(2\right)}\right\rangle$$

where χ^(2)^_R_ is the second-order susceptibility of a resonance, *N* is the number of oscillators contributing to the signal, ε_0_ is the dielectric permittivity, and ⟨β^(2)^_R_⟩ is the orientational average of the molecular hyperpolarizability. The sum frequency intensity *I*_VSF_ is proportional to the square of the effective second-order susceptibility χ^(2)^_eff_, including the Fresnel factors,^33^ and the intensities of the incoming beams, according to eq 2.

2 $$I_{VSF}\propto {\left|\chi_{eff}^{\left(2\right)}\right|}^{2}I_{vis}I_{IR}$$

The effective susceptibility is the product of the second-order susceptibility χ^(2)^ and the Fresnel factors. χ^(2)^ has a nonresonant part that predominantly originates from the substrate, and resonant parts originating from the adsorbate, as shown in eq 3.

3 $$\chi^{\left(2\right)}=\chi_{NR}^{\left(2\right)}+\sum_{n}\chi_{R,n}^{\left(2\right)}$$

All spectra have been fitted in accordance with eq 4,^10^ where the resonances are described by Lorentzian line shapes.

4 $$I_{VSF}\left(\omega_{IR}\right)\propto {\left|\chi_{NR,eff}^{\left(2\right)}+\sum_{n}\chi_{R,eff}^{\left(2\right)}\right|}^{2}\propto {\left|\chi_{NR,eff}^{\left(2\right)}+\sum_{n}\frac{A_{n}}{\omega_{n}-\omega_{IR}+i\Gamma_{n}}\right|}^{2}$$

where *A*_*n*_ is the amplitude, ω_*n*_ is the transition frequency, Γ_*n*_ is the damping constant of the *n*th vibration, and ω_IR_ is the frequency of the infrared laser beam.

## Experimental Section

The laser from Ekspla (Lithuania) with a pulse length of 27 ps, a repetition rate of 20 Hz, and a fundamental wavelength of 1064 nm was used to pump an OPG/OPA from Laservision (USA), generating a visible beam at 532 nm and a tunable infrared beam in the spectral region of 1000–4000 cm^–1^. The two beams are temporally and spatially overlapped at the surface and generate the sum frequency beam, which is detected by a monochromator, a photomultiplier tube (PMT), and a PC. A detailed description of the setup can be found elsewhere.^34^

All VSFS experiments were performed with a KSV minimicro 1S (Finland) Langmuir trough incorporated into the laser setup, as described earlier.^10^ Thus, VSFS data could be acquired in situ at the same time as the surface pressure and the molecular area were monitored. Since monolayers of unsaturated compounds may be susceptible to oxidation when exposed to laboratory air,^10^ all experiments were performed in a nitrogen atmosphere, which was accomplished by keeping the trough in a purged Plexiglass box. The experiments were performed at room temperature.

The ATR spectrum of GlcCer was obtained from GlcCer powder using a Bruker Tensor 37 with a diamond crystal and a spectral resolution of 2 cm^–1^.

## Materials

1,2-Distearoyl-*sn*-glycero-3-phosphocholine (DSPC), 1,2-distearoyl-d70-*sn*-glycero-3-phosphocholine-1,1,2,2-d4-N,N,N-trimethyl-d9 (DSPC-d83, hereafter denoted as d-DSPC), and d-glucosyl-ß-1,1′ *N*-stearoyl-D-*erythro*-sphingosine (GlcCer­(d18:1/18:0), hereafter denoted as GlcCer, all with a purity >99%, were purchased from Avanti lipids and used as received. Cholesterol (>99%) from Sigma Aldrich was used without further purification. All solutions were prepared using chloroform. Milli-Q water of 18.2 MΩcm was used as a subphase in the Langmuir trough. All molecules used are shown in Figure 1.

**Figure 1:**
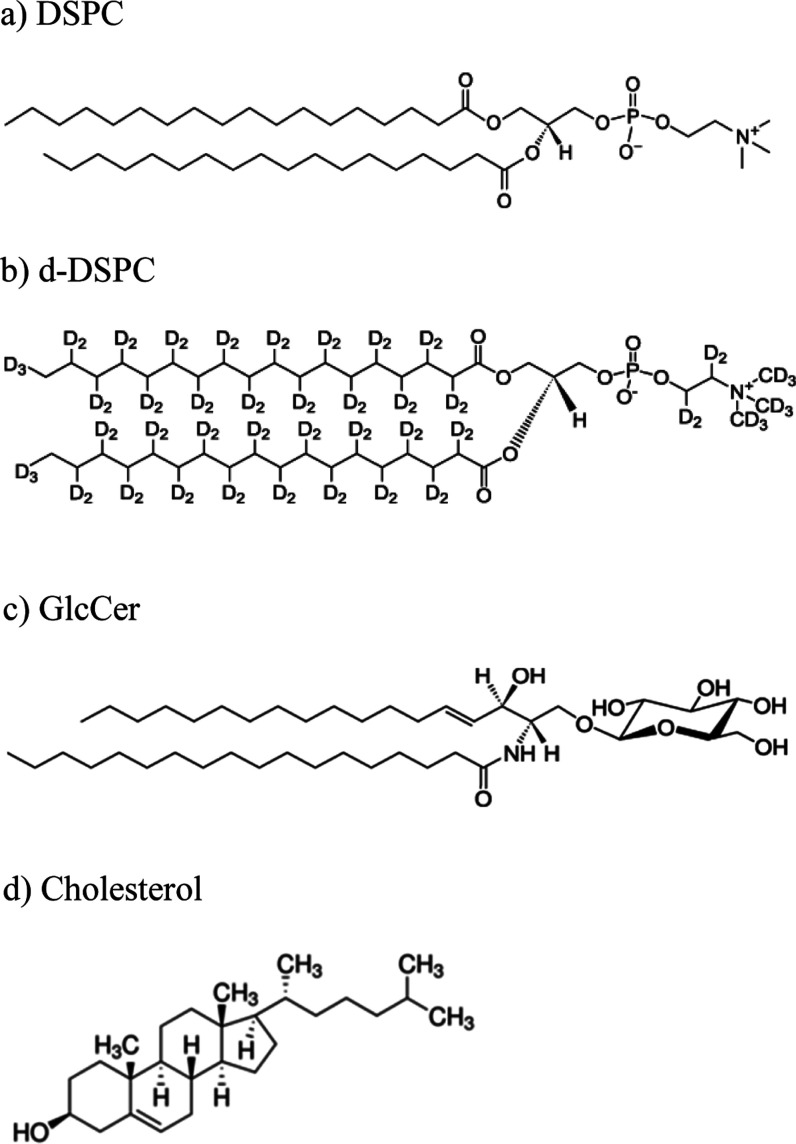
Molecules used in the experiments. (a) DSPC, (b) d-DSPC, (c) GlcCer, and (d) cholesterol.

## Results and Discussion

The target of the investigations is to determine how sphingolipids, saturated phosphatidylcholines, and cholesterol, all of them believed to be important constituents in lipid rafts, interact with each other as well as with the surrounding water. VSF spectra were acquired for individual molecules as well as mixtures thereof to scrutinize the molecular packing, orientation, and hydration of these biomolecules. The systems that have been studied are d-DSPC, GlcCer, cholesterol, d-DSPC/GlcCer, and d-DSPC/GlcCer/cholesterol. Spectra have been acquired at 4, 30, and 55 mN/m for GlcCer and at 30 mN/m for d-DSPC, cholesterol, the 50/50 mixture of GlcCer/d-DSPC, and the ternary 33/33/33 GlcCer/d-DSPC/cholesterol mixture, where 30 mN/m is close to the pressure in biological cell membranes.^35^ Thus, the studies allow a comparison of how the surface pressure affects the molecular structure. Detailed information about molecular interactions between these biomolecules and water can be obtained by comparing the spectra of lipid/cholesterol mixtures with the individual components.

VSF spectra in the CD, CH, and OH stretching regions of the biomolecules and water are reported. Thus, both the hydrocarbon tails as well as the hydration of the lipids were targeted independently. In general, the spectra of the mixtures have a lower signal-to-noise ratio than the individual components, simply because the number density of a certain lipid is lower. Equations 1 and 2 reveal that the sum frequency intensity is proportional to the square of the number of molecules, and thus, a strong dependency on the number density is found.

### GlcCer

VSF spectra of pure GlcCer at the air/water interface have been acquired at surface pressures of 4, 30, and 55 mN/m in the region of 2750–3850 cm^–1^, thus including the CH, NH, and OH stretches of the lipid, as well as the broad OH stretching region of hydrating water extending over basically the entire spectral region. The ssp, ppp, and sps polarization combinations were used in order to extract information about the order and orientation of the surface molecules at 30 mN/m. The data for 30 mN/m are shown below, whereas the complementary measurements at 4 and 55 mN/m are shown in the Supporting Information.

Figure 2 shows VSF ssp, ppp, and sps spectra in the CH stretching region at 30 mN/m, which corresponds to an area of 41 Å^2^/molecule. Identified peaks are the symmetric CH_2_ stretch at 2850 cm^–1^, the symmetric CH_3_ stretch at 2875 cm^–1^, the Fermi resonance between an overtone of the bending mode and the symmetric CH_3_ stretch at 2940 cm^–1^, and the antisymmetric CH_3_ stretch at 2965 cm^–1^.^36^ In addition, the congested region around 2900–2920 cm^–1^ contains CH_2_ Fermi resonances and the antisymmetric CH_2_ stretch.^36^ No signs of the vinyl CH stretch expected slightly above 3000 cm^–1^ are seen, neither in the ATR spectrum (Figure 3) nor in the VSF spectra. The ssp spectra are dominated by the symmetric CH_3_ stretch and the CH_3_ Fermi resonance, and the symmetric CH_2_ stretch and the antisymmetric CH_3_ stretch appear significantly weaker. In contrast, in the ppp and sps spectra, the antisymmetric CH_3_ stretch is strongest, and the symmetric CH_3_ stretch is weak.

**Figure 2:**
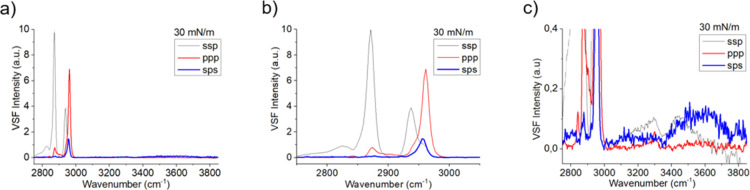
(a) VSF ssp, ppp, and sps spectra of GlcCer at 30 mN/m in the region of 2750–3850 cm^–1^, thus including the CH and OH stretching vibrations. (b) CH stretching region in (a) magnified; (c) OH region in (a) magnified. The maximum peak intensity of the symmetric methyl stretch has in each individual ssp spectrum been assigned a value of 10, and the corresponding ppp and sps spectra have been normalized with the same scaling factor to keep their relative intensities.

**Figure 3:**
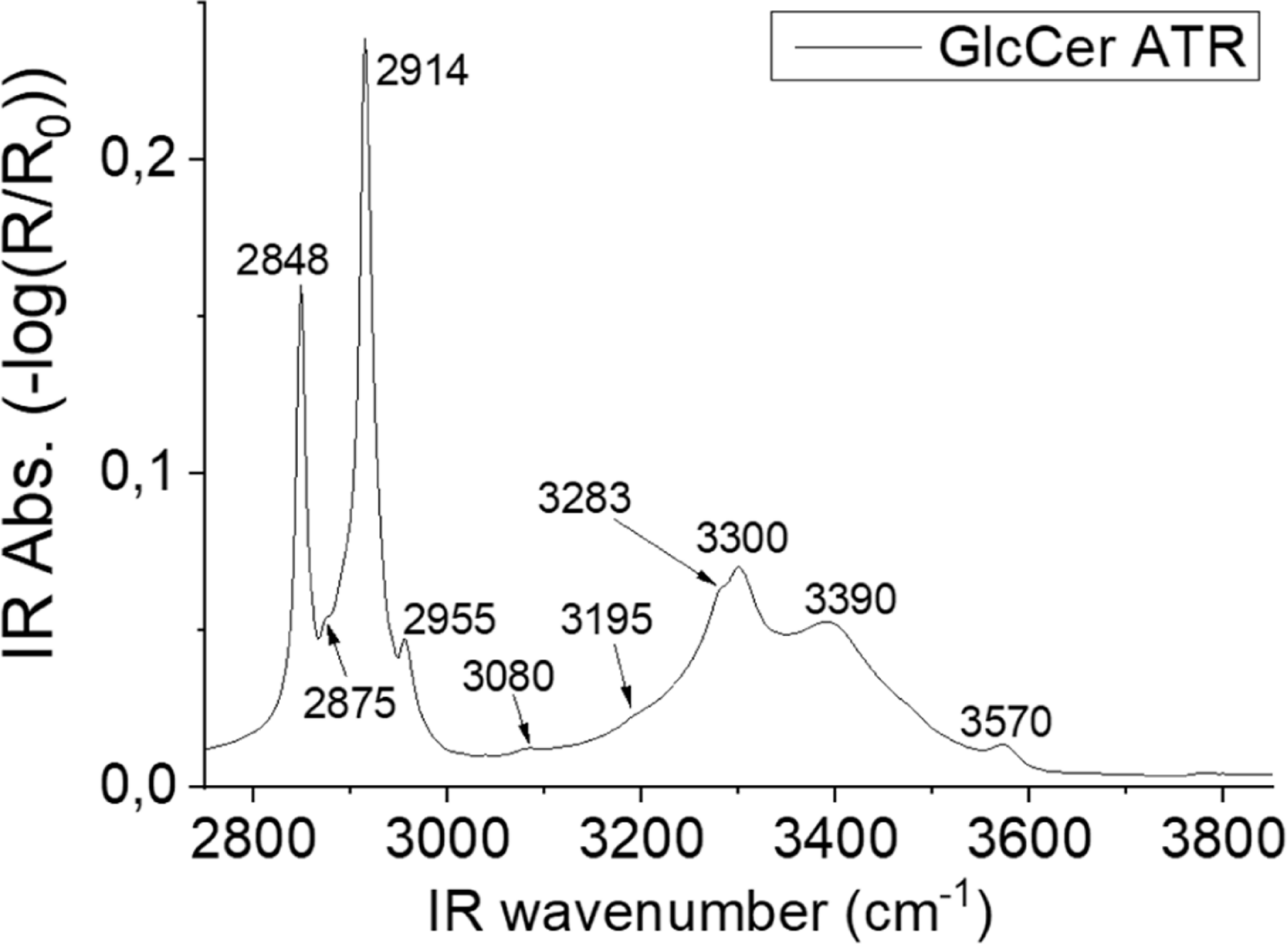
ATR spectrum of the powder of GlcCer.

In VSFS, the intensity ratio of the symmetric methyl stretch and the symmetric methylene stretch is often used to determine the molecular order of hydrocarbon chains.^37^ In chains with an all-trans configuration, there is an inversion center between each pair of methylene groups, and thus, they yield a zero-sum frequency signal.^38^ However, a methylene signal will be observed in the spectrum if gauche defects are present in the chains since the inversion center is no longer present. The terminating methyl group will always give rise to a sum frequency signal unless the system is completely disordered or the transition moment lies in the surface plane, since the symmetry around it is broken. Hence, a high CH_3_/CH_2_ intensity ratio will be observed for well-ordered hydrocarbon chains, whereas disordered systems result in a lower ratio.

As clearly seen in the ssp spectrum for 30 mN/m (and also in Figure S1 for 4 and 55 mN/m), the symmetric CH_3_ stretch at ∼ 2875 cm^–1^ is significantly more intense than the symmetric CH_2_ stretch at ∼2850 cm^–1^, an indication that the hydrocarbon chains are well packed. However, performing an unambiguous orientation analysis of the terminating CH_3_ groups is difficult,^9^ but a formal orientation analysis is performed for d-DSPC below, where the deuterated chains facilitate such an analysis.

Spectra in the OH stretching region of surface water molecules (∼3000–3800 cm^–1^) were acquired simultaneously as the CH region shown above, but since the OH bands are considerably weaker than the CH bands, the OH region is shown separately in Figure 2c. The reason for the low intensity in the OH stretching region is the fact that GlcCer is a neutral molecule and thus does not have the ability to order water molecules to the same extent as charged lipids.^39^ The charges create an electrical field at the interface, which enhances the order of the surrounding water molecules, leading to an increased VSF signal.^4^ In addition, the sugar group in GlcCer contains several different –OH groups with different orientations, and hence, the water molecules hydrating the sugar group likely also possess different orientations, which would lead to a reduction in the VSF signal. The spectra contain contributions from water molecules possessing different strengths of the hydrogen bonds (stronger hydrogen bonds at lower wavenumbers) as well as NH and OH stretches of the sphingolipid.^40–42^

The ssp spectrum displays a broad band over the whole region, which likely carries a contribution from water to a varying degree throughout the band. On the low-wavenumber side, two more distinct peaks at approximately 3290 and 3440 cm^–1^ are seen. A peak around 3300 cm^–1^ was observed in VSFS studies of surface protein layers on water and assigned to the N–H stretch,^27^ and a peak quite close, at 3283 cm^–1^, was observed for sphingomyelin in IR spectra.^42^ The ATR spectrum (Figure 3) of the powder of GlcCer shows a double peak at 3283 and 3300 cm^–1^, where one of the peaks likely belongs to the NH stretching vibration. The fairly sharp peak in the sum frequency spectra at 3290 cm^–1^ in Figure 2c (most clearly in ssp but also in sps) is hence assigned to the NH stretching vibration, although hydrating water molecules may contribute to this band as well.

In contrast to the ssp spectrum, the sps spectrum exhibits a low intensity up to around 3400 cm^–1^ and a broad band centered at approximately 3560 cm^–1^. Essentially similar features are observed in the ppp spectrum, although considerably weaker. Bands located at such high wavenumbers signify water molecules only participating in weak interactions and have been assigned to water in the proximity of hydrocarbon moieties in studies of surfactants possessing a sugar headgroup.^39^ However, a band at 3515 cm^–1^ has in IR studies been assigned to stretching vibrations of OH groups of the sphingolipid itself, participating in very weak hydrogen bonding.^41^ The broad band in the ppp and sps VSF spectra can thus originate from both water molecules participating in weak interactions, as seen for other small organic molecules^39,43–45^ as well as the OH groups of GlcCer.

### Cholesterol

VSF spectra have been acquired at 30 mN/m in the region of 2750–3850 cm^–1^ for cholesterol, and several intense peaks in the CH stretching region show up as seen in Figure 4, revealing that cholesterol forms an ordered layer at the water surface. The peaks observed are at 2817 cm^–1^ (unassigned), the symmetric CH_2_ stretch at 2847 cm^–1^, the symmetric CH_3_ stretch at ∼2870 cm^–1^, the R_3_C–H stretch at 2907 cm^–1^, the CH_2_ antisymmetric stretch and CH_2_/CH_3_ Fermi resonances at 2940 cm^–1^, and the antisymmetric CH_3_ stretch at ∼2960 cm^–1^.^15,21,36^ Since no further analysis is performed using these peaks, the wavenumbers are based on peak maxima rather than on curve fits.

**Figure 4:**
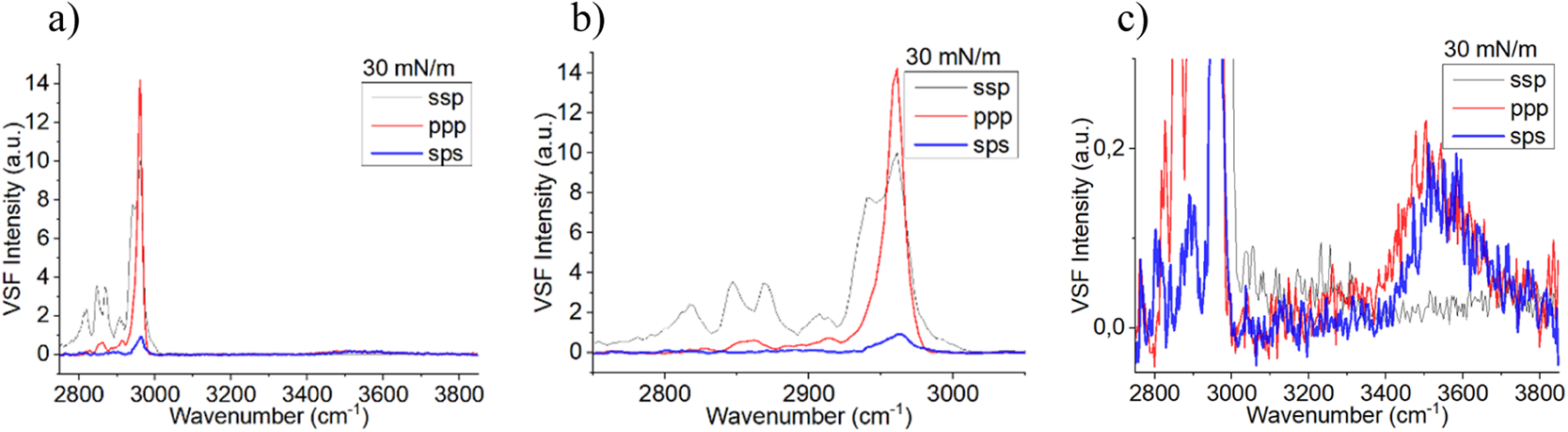
(a) VSF spectra of cholesterol at 30 mN/m at the air/water interface in the (a) CH/OH stretching regions, (b) the spectra in (a) magnified to better visualize the CH region, and (c) the spectra in (a) magnified to better visualize the OH region.

It should be noted that the peaks bear contributions from several different CH/CH_2_/CH_3_ groups located at various positions in the cholesterol molecule (Figure 1), hence experiencing different local environments and orientations. The VSF spectra are thus a weighted average of all different groups, and a further analysis of, for example, the orientation of a certain functional group does not carry any relevance. A consequence of the fact that several different methyl groups (five methyl groups in cholesterol, of which at least four of them are different) contribute to the signal is that the antisymmetric methyl stretch at ∼2960 cm^–1^ is more intense than the symmetric methyl stretch at ∼2870 cm^–1^ in the ssp spectrum, which is not observed for simple lipids or surfactants, for example, as seen for GlcCer above. However, a similar observation was done for the anesthetic drug propofol in contact with phospholipid monolayers as well as at the air/water interface.^46,47^ Similarly to cholesterol, propofol possesses several methyl groups, and the weighted average orientation results in a more intense antisymmetric methyl stretch compared to the symmetric stretch.

Due to the hydrophilic nature of the OH group in cholesterol, it is assumed to form hydrogen bonds with water, and the hydrocarbon parts are directed toward the air. The bands in the region of ∼3000–3850 cm^–1^ in the ssp spectrum originate from water molecules hydrating the OH group in cholesterol and the OH group itself. Certainly, water hydrates the OH group of cholesterol, but the reason for the very weak water intensity in the ssp spectrum essentially over the whole spectrum is the fact that cholesterol is uncharged and cannot align water molecules because there is no strong electric field in the surface region. Further, the low intensity of the band may be due to a broad orientational distribution of water molecules. In contrast to the very weak and featureless water bands observed in the ssp spectrum, the bands in the ppp and sps spectra centered around 3500–3550 cm^–1^ are more pronounced and originate from water molecules weakly interacting with neighbors. Similar bands have been assigned to water in the proximity of the hydrocarbon chains, which also applies here.^39^ A negligible intensity in ssp and intense bands in ppp and sps indicate that the origin of the band at 3500 cm^–1^ is water molecules with C_2v_ symmetry participating in stretching vibrations with an asymmetric character.^39^

### d-DSPC

As seen in Figure 1, d-DSPC is not fully deuterated but contains two CH_2_ groups and a single CH group, which will contribute to the signal in the CH stretching region.^9^ Figure 5 shows the ssp, ppp, and sps spectra for d-DSPC at a surface pressure of 30 mN/m on a water surface.

**Figure 5:**
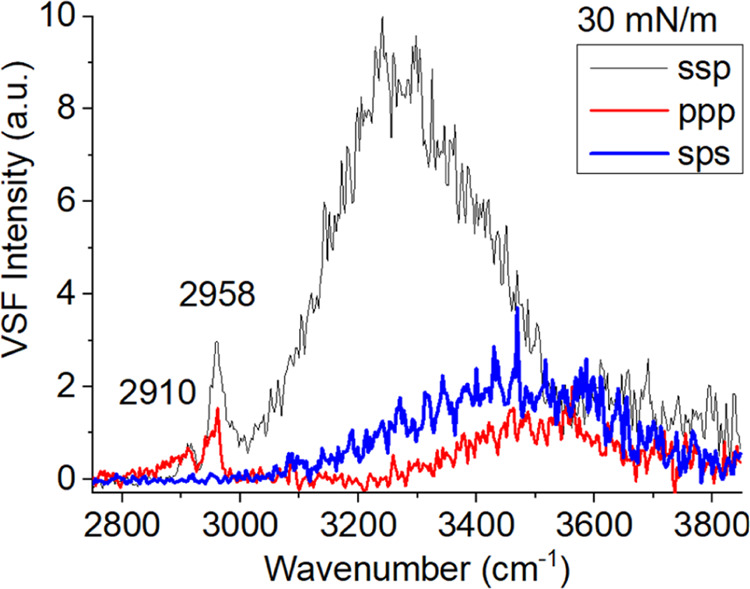
VSF spectra of d-DSPC acquired under the ssp, ppp, and sps polarization combinations at 30 mN/m. The highest water peak in ssp has arbitrarily been normalized to 10 (in other spectra, the symmetric CH_3_ stretch has been normalized to 10, but there is no CH_3_ group in d-DSPC), and the ppp and sps spectra have been normalized with the same scaling factor.

In both the ssp and ppp spectra, peaks at around 2910 and 2960 cm^–1^ are observed, which is in agreement with studies of the same molecules adsorbed onto CaF_2_ substrates.^9^ Thus, in addition to GlcCer and cholesterol, d-DSPC will also contribute to the VSF signal in the CH stretching region for VSF spectra of mixtures of these molecules. However, comparing the relative intensity of the CH peaks to the broad water band centered at ∼ 3300 cm^–1^ for d-DSPC and nondeuterated DSPC^11^ reveals that the contribution from d-DSPC in the CH region is considerably weaker than for DSPC (and hence also compared to GlcCer and cholesterol) as the broad water band essentially will be unaffected by the deuteration of the hydrocarbon chain. Thus, to a good approximation, the contribution to the spectrum in the CH stretching region is negligible for d-DSPC. The spectra of d-DSPC in the CD stretching region are discussed together with the spectra of the mixtures with cholesterol and GlcCer to facilitate a comparison of the molecular structure of d-DSPC alone and in the mixtures. The hydration of DSPC has been studied earlier, and the spectra presented here are in agreement with earlier studies.^11^ In comparison with GlcCer and cholesterol, the water bands are significantly more intense, a result of the fact that DSPC is charged and hence can orient more water molecules.

### Mixtures of d-DSPC, GlcCer, and Cholesterol

VSF spectra of mixtures of the compounds were acquired in the CD, CH, and OH stretching regions. The focus has been on the CD stretching region of d-DSPC since the CH stretching region is difficult to study in detail as all molecules contain CH_*x*_ groups.

The order and orientation of unmixed d-DSPC were compared with a mixture of 50/50 d-DSPC/GlcCer and a mixture of 33/33/33 d-DSPC/GlcCer/cholesterol to investigate how the molecular packing of d-DSPC is affected by the other biomolecules in simulated lipid rafts. For all systems, spectra were acquired for the ssp, ppp, and sps polarization combinations at a surface pressure of 30 mN/m, as shown in Figure 6. Since lipid rafts are known to be enriched in saturated phospholipids, sphingolipids, and cholesterol, this step-by-step investigation of the molecular structure provides detailed information about how these important biomolecules interact with themselves as well as other constituents in rafts. Since the VSF signal is proportional to the square of the number of molecules according to eqs 1 and 2, the strength of the signal will drastically decrease for the mixtures in comparison with the pure compounds. Hence, the signal in Figure 6c is very weak in comparison to the spectra in 6a and 6b. For all three systems, the symmetric CD_3_ stretch at 2075 cm^–1^ dominates the ssp spectra, its Fermi resonance (FR) is observed at 2130 cm^–1^, and the antisymmetric CD_3_ stretch is observed at 2225 cm^–1^.^48^ The symmetric and antisymmetric CD_2_ stretches at 2100 and 2195 cm^–1^, respectively, are essentially absent. In the ppp spectra, the antisymmetric CD_3_ stretch is dominant and the antisymmetric CD_2_ stretch is observed as a weak shoulder. In the sps spectra, only the antisymmetric CD_3_ stretch can be clearly distinguished.

**Figure 6:**
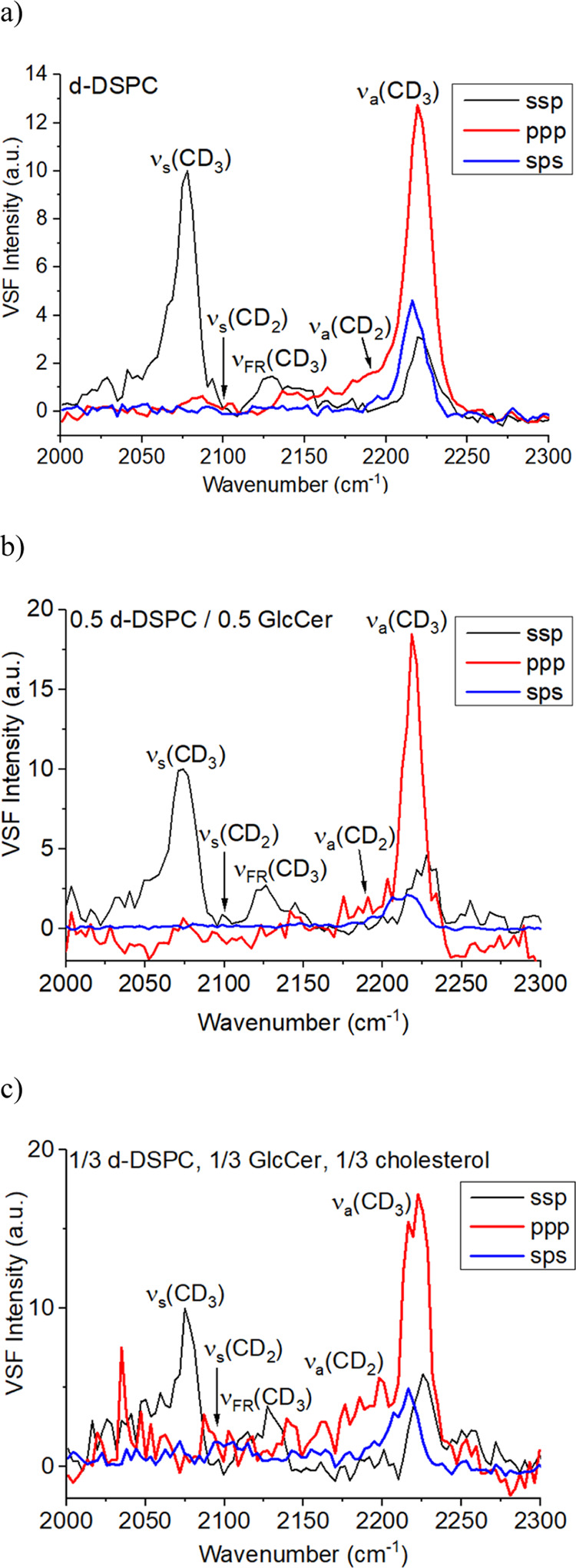
Sum frequency spectra under the ssp, ppp, and sps polarization combinations in the CD stretching region for (a) d-DSPC, (b) a 50/50 mixture of d-DSPC and GlcCer, and (c) a mixture of 1/3 of each of the components d-DSPC, GlcCer, and cholesterol. In each graph, the ssp intensity of the symmetric CD_3_ stretch has been normalized to 10, and the ppp and sps polarizations accordingly. All spectra have been acquired at a surface pressure of 30 mN/m.

In contrast to the commonly used r^+^(CH_3_)/r^+^(CH_2_) amplitude ratio to estimate the chain order (the presence or absence of gauche defects),^10,37^ for deuterated chains, it has been proposed that the amplitude ratio of the antisymmetric CD_3_ stretch at 2220 cm^–1^ and the antisymmetric CD_2_ stretch at ∼2190 cm^–1^ in ppp should be used instead.^48^ This distinction is based on the fact that the symmetric CD_2_ stretch has a very low cross-section and is thus unsuitable for such an analysis. In the spectra of all three systems, the CD_3_ stretch is appreciably more intense than the antisymmetric CD_2_ stretch in ppp, hence indicating that the d-DSPC chains are well packed in all systems. This supports the idea that lipid rafts, which are enriched in saturated phospholipids, sphingolipids, and cholesterol, are well-ordered structures with chains having few gauche defects, which contrasts with the other parts of the plasma membrane consisting of more disordered unsaturated phospholipids.^10^ The antisymmetric CD_2_ stretch at 2190 cm^–1^ is possibly slightly more intense for the triple mixture, but the low signal-to-noise ratio does not allow a clear conclusion to be made.

### Orientation Analysis of Hydrocarbon Tails of d-DSPC

An orientation analysis has been performed for the terminating – CD_3_ group for pure d-DSPC at 30 mN/m and for the same group in the different mixtures with GlcCer and cholesterol. No such analysis could be performed for GlcCer in the ternary mixtures since the CH peaks originating from cholesterol overlap with the GlcCer peaks.

There are several possibilities to perform an orientation analysis for methyl groups using VSFS. Here, we assume the surface plane to be isotropic, that the methyl group has C_3v_ symmetry, and that it can rotate freely. In some studies, the lower *C*_s_ symmetry of the methyl group has been used, but since the antisymmetric methyl stretch in these spectra could be accurately fitted with a single resonance, *C*_3v_ has been used. Moreover, for only a slight splitting of <10 cm^–1^ of the two antisymmetric stretches, the error introduced by assuming a degenerate vibration in the orientation analysis is estimated to be low.^49,50^ The polarization combinations that have been used in the analysis are ssp and sps, since they only depend on a single element of the nonlinear susceptibility tensor (yyz for ssp and yzy for sps), in contrast to the ppp polarization combination, which relies on three independent tensor elements. Another common method has been to use the symmetric methyl stretching vibration in the orientation analysis. However, this method requires the β_aac_/β_ccc_ ratio to be known, whereas no hyperpolarizability ratios are needed when using the antisymmetric stretch. Thus, we conclude that the antisymmetric stretch is most reliable for an orientation analysis of the −CD_3_ group in this case.^48^ In the analysis, the sum frequency, visible, and infrared refractive indices for air have been set to 1, for the subphase they have been set to the values of pure water (*n*_SF_ = 1.336, *n*_vis_ = 1.334 *n*_IR_ = 1.332 + 0.0134i), and the interfacial refractive indices have all been set to 1.2, which has been determined to be a proper value for terminating methyl groups.^51^ A thorough description of how to perform an orientation analysis is given in ref 9.

Figure 7 shows the calculated *A*_ssp_/*A*_sps_ curves for the antisymmetric stretch of the terminating CD_3_ group of d-DSPC for pure d-DSPC (curve fits in Figure S1) and the mixtures 50/50 d-DSPC/GlcCer (curve fits in Figure S2) and 33/33/33 d-DSPC/GlcCer/cholesterol (curve fits in Figure S3). All fitting parameters are found in Table S1 in the Supporting Information. The curve represents a δ-distribution of tilt angles. However, a more physically relevant condition would be to assume a Gaussian distribution of the tilt angle, but since the main interest in this study is to make a comparison of the tilt angle between different mixtures, only the δ-distribution has been used. The horizontal shaded areas and the line represent the amplitude ratio intervals, and the vertical lines represent the corresponding tilt angle ranges. The data is based on curve fits (Figure S1 for pure d-DSPC, Figure S2 for the mixture 50/50 d-DSPC/GlcCer, and Figure S3 for the mixture 33/33/33 d-DSPC/GlcCer/cholesterol), which are shown together with the corresponding fitting parameters in Table S1 in the Supporting Information. All fits are decent, especially in the region around 2220 cm^–1^, where the antisymmetric CD_3_ stretch that is used in the analysis is observed. The tilt angle of the terminating CD_3_ group in d-DSPC lies in the interval of 36–41° for pure d-DSPC, 46–47° for the CD_3_ group in the 50/50 mixture of d-DSPC/GlcCer, and 44° for the CD_3_ group in the 33/33/33 d-DSPC/GlcCer/cholesterol mixture. Since the signal in the case of the ternary mixture was extremely weak (see above), only the fits from one experiment resulted in trustworthy fitting parameters, and hence, a horizontal line is shown instead of a range. However, the spectra from different days qualitatively resembled each other, and thus, the line represents a reasonable value. Since the tilt angle of the hydrocarbon chain (α) is related to the methyl tilt angle by the equation α = 41.5 – θ for an all-trans configuration,^52^ the chain tilt angle will be close to zero, and hence, the d-DSPC molecules basically stand straight up. Using the extreme tilt angles of the methyl groups for all three systems, the maximum deviation is 11°, and the minimum deviation is 6° (41–47°). This demonstrates that the molecular packing does not change appreciably when comparing pure d-DSPC with d-DSPC in mixtures with the other compounds enriched in lipid rafts. Hence, these results are in line with the idea that saturated phospholipids, sphingolipids, and cholesterol can form well-ordered lipid rafts in cell membranes.

**Figure 7:**
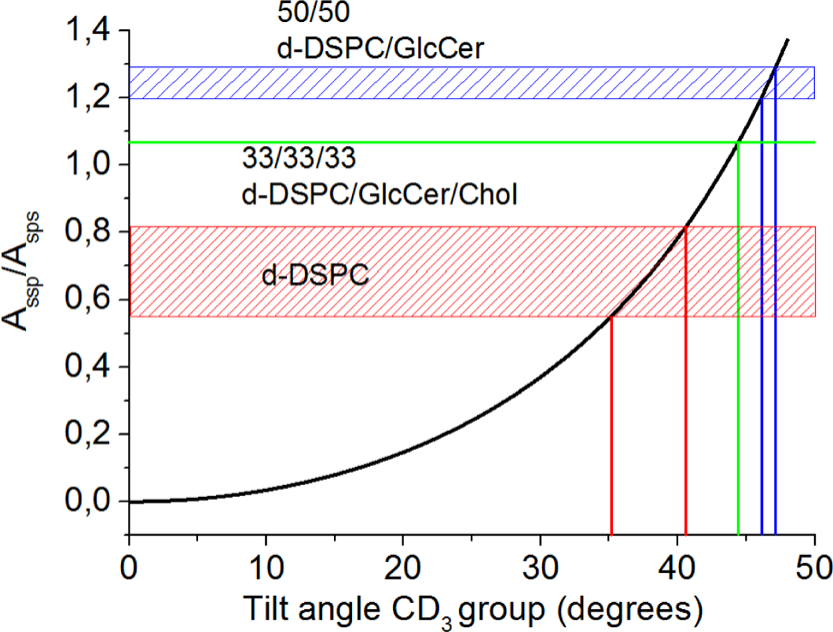
Theoretical graphs showing the tilt angle of the CD_3_ group in d-DSPC as a function of the amplitude ratio *A*_ssp_/*A*_sps_ for pure d-DSPC, a 50/50 mixture of d-DSPC/GlcCer, and a 33/33/33 mixture of d-DSPC/GlcCer/cholesterol. The horizontal ranges indicate the experimental spread in the amplitude ratio, and the vertical lines show the corresponding tilt angles.

In an earlier study, the orientation of the CD_3_ group of an LB film of d-DSPC on a CaF_2_ substrate was determined to be 37°,^9^ thus similar to the case of pure d-DSPC on water in this study. Hence, it appears that the different types of substrates (water and solid CaF_2_) do not affect the orientation of the chains.

### CH and OH Stretching Regions of Mixed Systems

The presence of GlcCer in the 50/50 mixture with d-DSPC is revealed by a strong signal in all polarization combinations in Figure 8a,b. The high r^+^(CH_3_)/r^+^(CH_2_) ratio signifies that the GlcCer molecules are highly oriented, with only a low number of gauche defects. Hence, in the mixture of GlcCer with d-DSPC, the high order of the GlcCer molecules observed for pure GlcCer above is retained, similar to that for d-DSPC, as shown in Figure 6. The CH stretching region for the triplet mixture in Figure 8d,e shows several peaks. The CH stretching region has also been magnified in Figure S4 for the triplet mixture, where the spectra of different pure compounds as well as mixtures in each graph have been adjusted to similar intensities to make comparisons easier. From Figure 6, the presence of d-DSPC in the surface region is obvious, whereas its contribution to the spectra in the CH stretching region is low, as revealed by its weak signal in Figure 5. A detailed comparison of peaks that also reveals the presence of cholesterol at the surface is shown in Figure S5, and as discussed in the Supporting Information the presence of GlcCer at the surface is expected although it does not possess any specific functional groups not present in the other molecules that gives rise to peaks in the CH stretching region.

**Figure 8:**
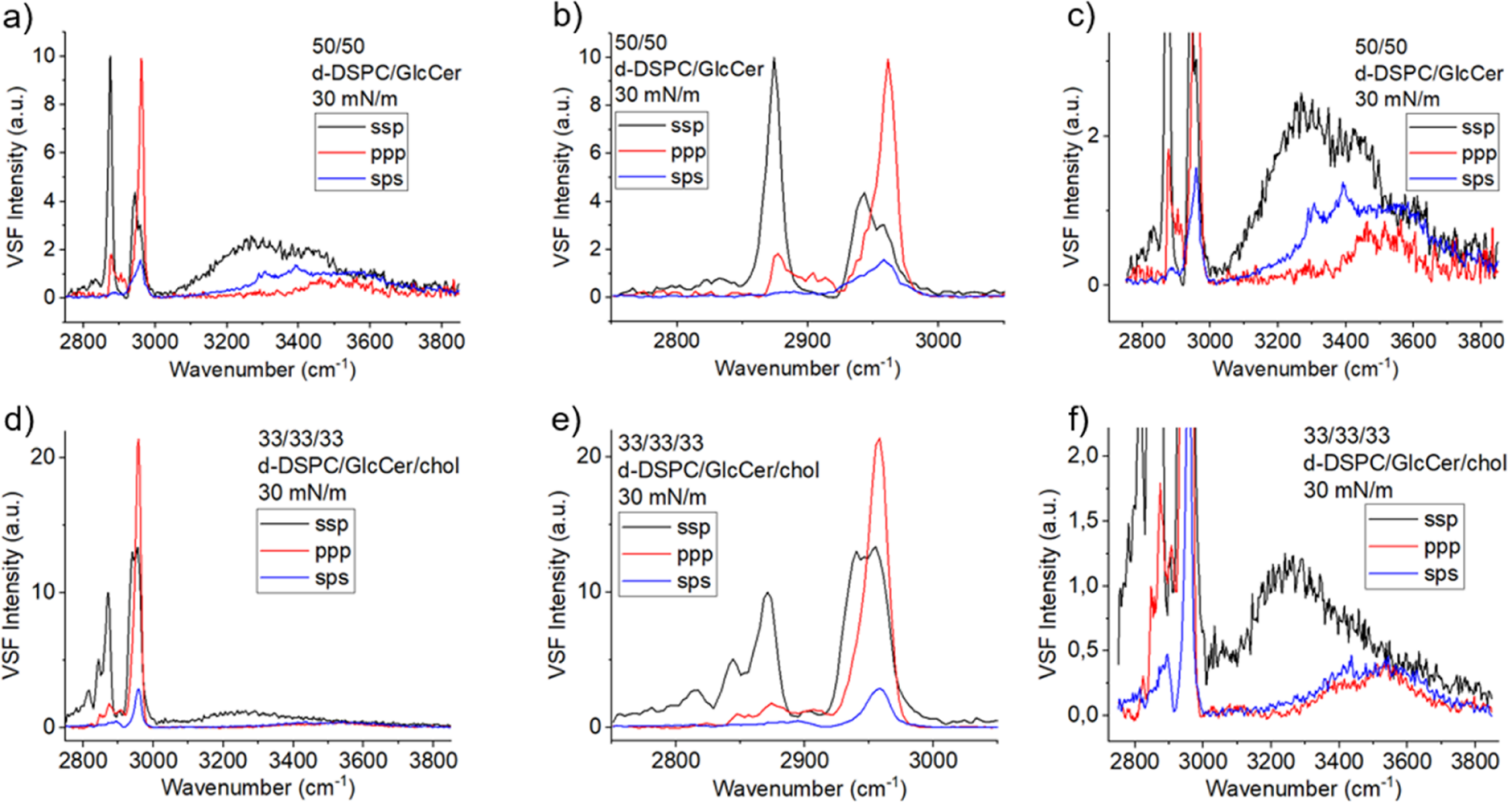
VSF spectra acquired at all three polarization combinations at 30 mN/m for (a–c) the 50/50 mixture of d-DSPC and GlcCer where the spectra in the left column have been magnified in the CH region in the middle column and in the OH region in the right column and for (d–f) the ternary mixture of d-DSPC, GlcCer, and cholesterol, where the spectra in the left column have been magnified in the CH region in the middle column and in the OH region in the right column.

The water molecules residing at the surface give rise to broad bands in the region of ∼3000–3800 cm^–1^. In addition, GlcCer, d-DPSC, and cholesterol contain OH groups, the stretching vibrations of which may contribute to the total sum frequency signal, and there is a further NH group present in GlcCer that has a stretching vibration overlapping with the water region. Thus, the fact that several functional groups contribute to the VSF signal and that the signal depends on both the number of molecules and their average orientation makes it essentially impossible to quantify and compare the amount of water molecules hydrating the biomolecules in the pure and mixed systems. However, it is possible to compare the relative intensities in ssp, ppp, and sps for the different systems, as well as the shape of the bands, which is done in Figure 8.

As seen in Figure 8a,c (in Figure 8c, the water region in Figure 8a is zoomed in) for the 50/50 mixture of d-DSPC and GlcCer, the broad band from ∼3000–3800 cm^–1^ is relatively strong in comparison with the CH_*x*_ peaks and the shape resembles the shape of the band for d-DSPC in Figure 5. Being charged, d-DSPC can orient more interfacial water molecules than the neutral molecule GlcCer, and hence, d-DSPC should to a larger extent influence the shape of the band, as observed. Moreover, the ppp spectrum resembles the spectrum of d-DSPC. The sps spectrum is dominated by the broad water band, but due to a low signal-to-noise ratio, the exact features are not entirely discernible. In Figure 8d,f for the triplet mixture, the water bands are significantly weaker in comparison with the same bands in Figure 8a,c for the 50/50 mixture, a result of the fact that the fraction of d-DSPC, the molecules that mostly contribute to ordering water, is lower. However, the overall band shapes for the three polarization combinations resemble the shapes in Figure 5 for d-DSPC, revealing that d-DSPC dominates when it comes to ordering the water.

## Conclusions and Outlook

In this article, the order, orientation, and hydration of the three compounds d-DSPC, GlcCer, and cholesterol at the air/water interface have been studied by VSFS at various surface pressures. At surface pressures of 4, 30, and 55 mN/m, the hydrocarbon chains of GlcCer were well ordered as revealed by a significantly more intense ssp signal for the symmetric methyl stretch in comparison with the symmetric methylene stretch. Similarly, d-DSPC was shown to form a highly ordered monolayer at the water surface at a surface pressure of 30 mN/m. In mixtures of these compounds, more specifically of a 50/50 mixture of d-DSPC and GlcCer and a 33/33/33 mixture of d-DSPC, GlcCer, and cholesterol, d-DSPC kept a well-ordered structure. The results regarding this model system of lipid rafts are thus in agreement with the idea that natural lipid rafts in cell membranes rich in saturated phospholipids, cholesterol, and sphingolipids can form highly ordered layers. Using the three polarization combinations ssp, ppp, and sps, it was concluded that the orientation of the terminating -CD_3_ groups in d-DSPC exhibited a tilt angle in the range of 36–47° at a surface pressure of 30 mN/m in all systems studied. Hence, the orientation remains fairly constant when going from pure d-DSPC to the 50/50 and 33/33/33 mixtures. The three types of molecules have a different ability to orient surrounding water molecules, and whereas the neutral molecules cholesterol and GlcCer only affect a low amount of water molecules, the charged d-DSPC can align more layers of water molecules. The spectra of the mixtures in the water stretching region around 3000–3800 cm^–1^ mostly resemble the profile of pure d-DSPC, thus indicating that d-DSPC dominates the effect of aligning water molecules.

The VSFS results obtained in these studies originate from probing areas hundreds of μm in diameter, thus considerably larger than what lipid rafts are assumed to be. It would therefore be of interest to employ near-field techniques such as nano-FTIR spectroscopy on the same systems as in this article, to probe areas around 20 nm in diameter.^53^ Such studies could reveal more about the local packing of biomolecules present in cell membranes, especially if, for example, unsaturated phospholipids that are not present in lipid rafts were included, since it would allow examinations of domain formation on the nano-level.

## Supplementary Material



## Notes

Curve fits and fitting parameters for the spectra used in the orientation analysis, as well as spectra magnified in the CH stretching region (PDF)
