# Supplementary material for: Molecular Ordering and Hydration in Model Lipid Rafts Studied by Vibrational Sum Frequency Spectroscopy
Source: J Phys Chem B. 2026 Jun 26;130(27):6816–25. doi: 10.1021/acs.jpcb.6c01062 (PMC13359109; doi:10.1021/acs.jpcb.6c01062)
Supplement: Supplementary file 1 [file jp6c01062_si_001.pdf]

Supporting information to

Molecular Ordering and Hydration in Model Lipid Rafts studied by Vibrational Sum Frequency Spectroscopy

Jonas Hedberg[1], C. Magnus Johnson[2]*

[1] Now at Surface Science Western, The University of Western Ontario, London, Ontario N6G 0J3, Canada (earlier same as [2])

[2]Division of Surface and Corrosion Science, Department of Chemistry, KTH Royal Institute of Technology, SE-100 44 Stockholm, Sweden.

To examine how the surface pressure affects the properties of the GlcCer monolayer, experiments have been performed at three different pressures, and figure S1 shows the ssp, ppp, and sps spectra of GlcCer at 4, 30, and 55 mN/m, with corresponding molecular areas of 44, 41, and 38 $Å^2$. As seen in the figures S1 a, c, e, the CH_2 intensity is relatively speaking slightly larger compared to the symmetric CH_3 stretch at 2875 cm^{-1} at 4 mN/m (the symmetric CH_3 stretch at ~2875 cm^{-1} is normalized to 10 in all spectra) compared to 30 and 55 mN/m. Thus, this indicates that the hydrocarbon chains are slightly more disordered at 4 mN/m.[33] This can be explained by the fact that the higher molecular area at 4 mN/m allows the chains to be more flexible. Further, the orientation of the terminating methyl groups appears to be essentially constant with surface pressure, as revealed by the similar relative peak intensities in the ssp, ppp, and sps spectra for the three surface pressures.

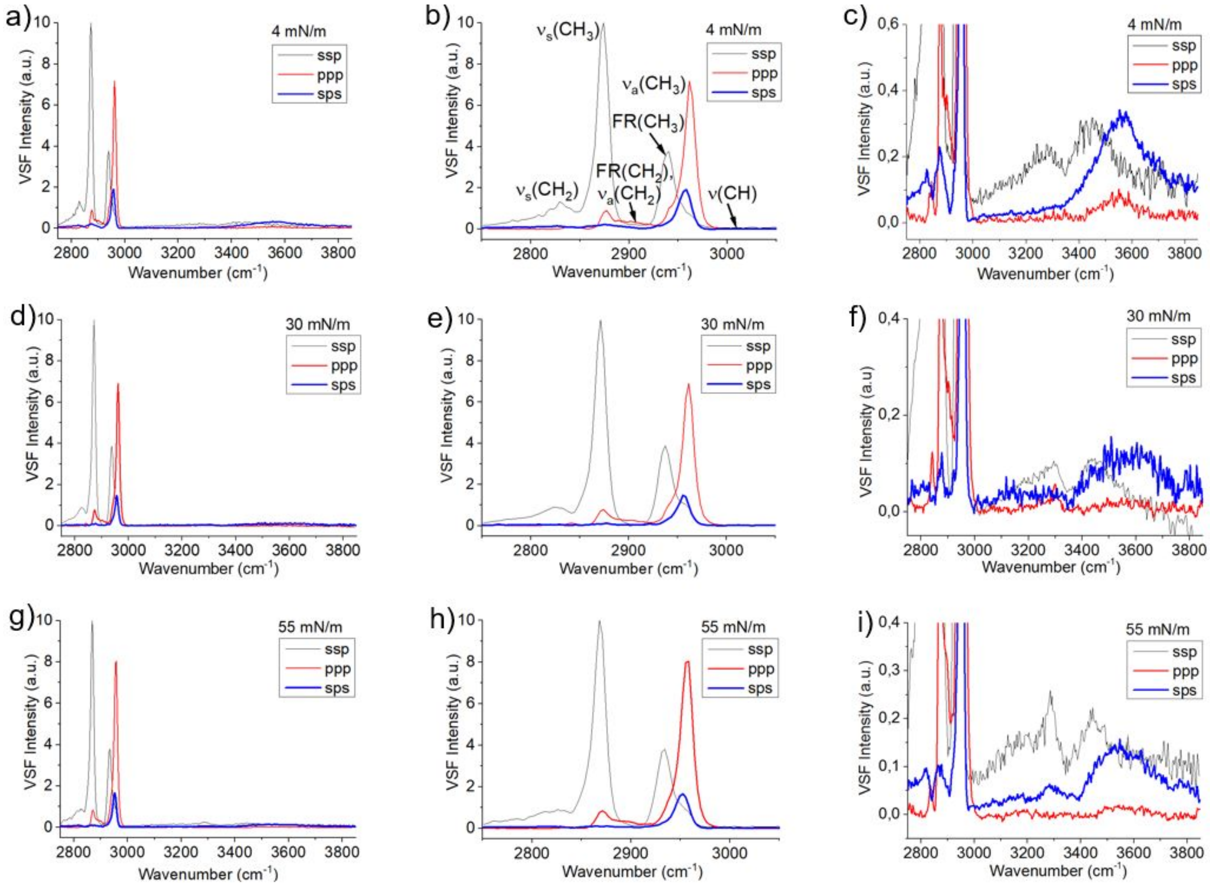

Figure S1: VSF spectra of GlcCer in the region 2750-3850 cm^{-1}. The graphs show the ssp, ppp, and sps spectra of GlcCer at a), b), c) 4 mN/m in the CH and OH stretching regions, d), e), f) 30 mN/m in the CH and OH stretching regions, and g), h), i) 55 mN/m in the CH and OH stretching regions. The middle column shows the left column magnified in the CH region and the right column shows the left column magnified in the OH region. In each spectrum, the maximum peak intensity of the symmetric methyl stretch has been assigned an arbitrarily value of 10. Accordingly, to keep the relative peak intensities, the corresponding ppp and sps spectra have been normalized with the same scaling factor. The spectra at 30 mN/m are the same as in the article but are included here as well to facilitate a comparison between the different surface pressures.

Below are the fits and the corresponding fitting parameters used for the orientation analysis of the terminating -CD_3 group in the different mixtures shown.

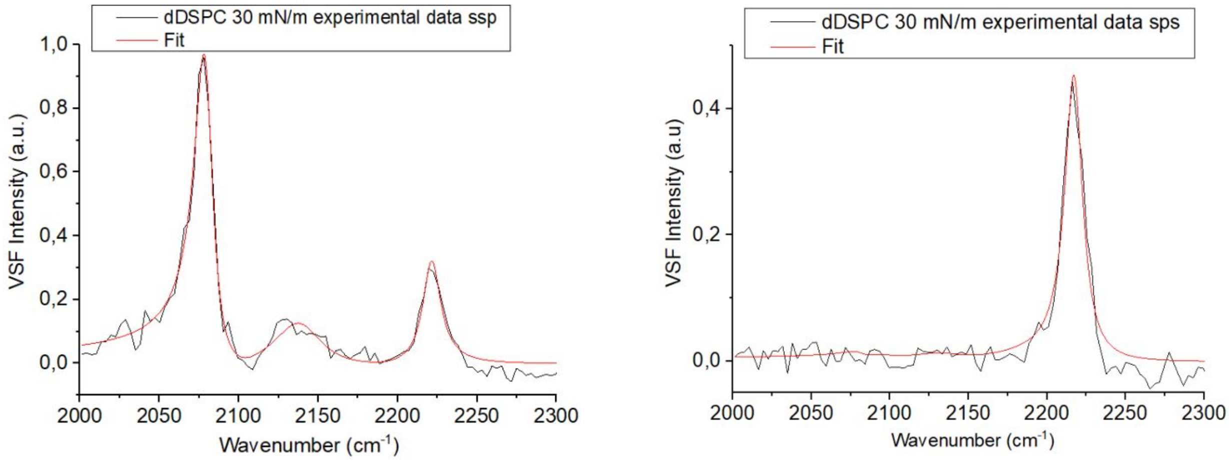

Figure S2: The fit for dDSPC at 30 mN/m.

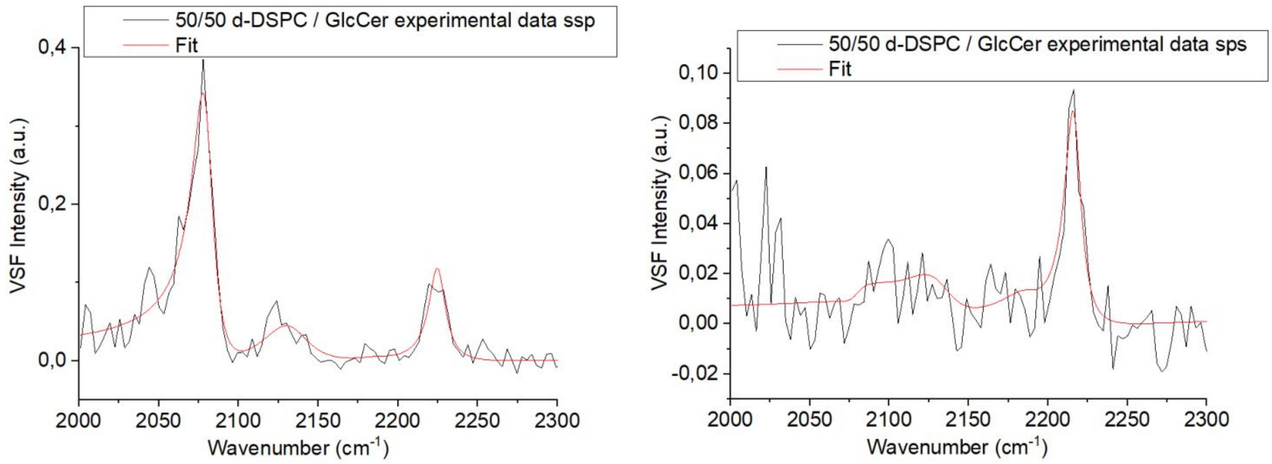

Figure S3: The fit for the 50/50 mixture of dDSPC / GlcCer at 30 mN/m.

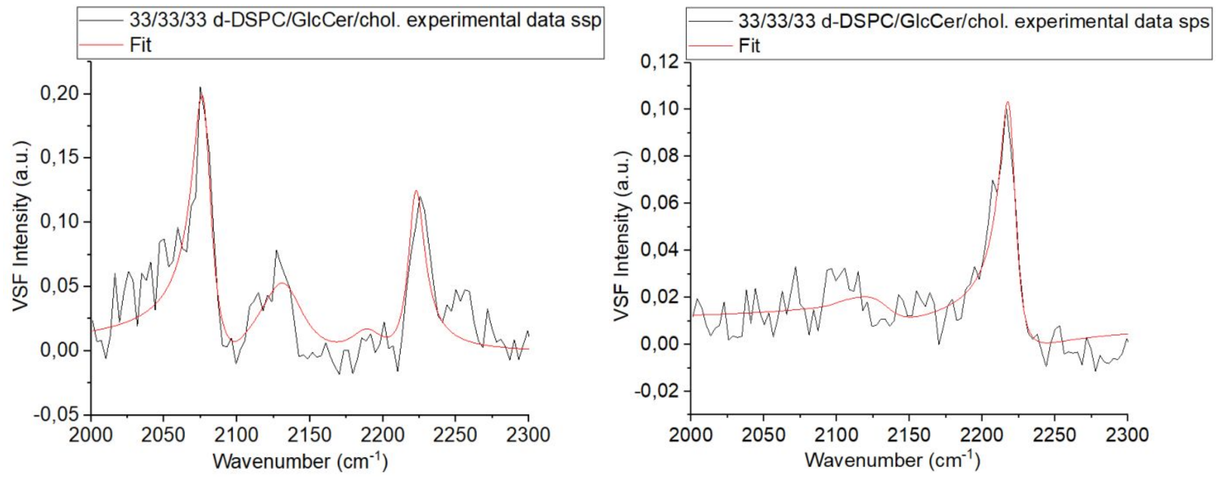

Figure S4: The fit for the 33/33/33 mixture of dDSPC / GlcCer /cholesterol at 30 mN/m.

The fitting parameters for the fits in figure S2-S4 are found in table S1. The fitted bandwidths have been restricted to the same value for each sample and the value of the imaginary nonresonant part has been set to zero in all fits. The peak wavenumbers were allowed to vary in a range of around 6 cm^{-1}.

Table S1: Fitting parameters for the VSF spectra.

**Fitting parameter**	**d-DSPC ssp**	**d-DSPC sps**	**50/50 d-DSPC/ GlcCer ssp**	**50/50 d-DSPC/ GlcCer sps**	**33/33/33 d-DSPC/ GlcCer/ chol ssp**	**33/33/33 d-DSPC/ GlcCer/ chol sps**
**A1**	6.6±0.089	0.27±0.76	4.2±0.22	-0.18±0.55	3.2±1.5	0±0.16
**ω1**	2079	2081	2080	2079	2078	2081
**Γ1**	7.2	7.2	8	8	8	8
**A2**	-0.2±0	0.26±0.89	-0.1±0	0.1±0	-0.2±2.8	-0.04±0.2
**ω2**	2105	2099	2099	2100	2099	2099
**Γ2**	10	10	15	15	10	10
**A3**	6.4±0.33	0.83±0.67	3.6±0.46	1.5±0.6	4.0±0.6	0.8±0.25
**ω3**	2139	2139	2133	2133	2133	2133
**Γ3**	19	19	19	19	19	19
**A4**	-0.2±0	0.09±0	0.32±0.72	0.52±0.57	1.3±0.5	0±0.24
**ω4**	2195	2188	2188	2188	2190	2195
**Γ4**	14	14	14	14	14	14
**A5**	3.8±0.20	4.6±0.11	2.1±0.14	1.1±0.16	2.2±0.5	2.0±0.06
**ω5**	2221	2217	2225	2217	2222	2219
**Γ5**	6.9	6.9	6.4	6.4	6.9	6.9
**NR Re**	0.096±0.010	0.047±0.01	0.09±0.01	0.07±0.014	0.04±0.02	0.1±0.005
**NR Im**	0	0	0	0	0	0

From the spectra in figure S5, the presence of cholesterol is observed. Cholesterol is revealed by a peak at approximately 2815 cm^{-1} since the other molecules do not show any vibrations there. GlcCer does not possess a peak not observed for the other molecules but is assumed to be present at the surface due to its long hydrophobic hydrocarbon tails.

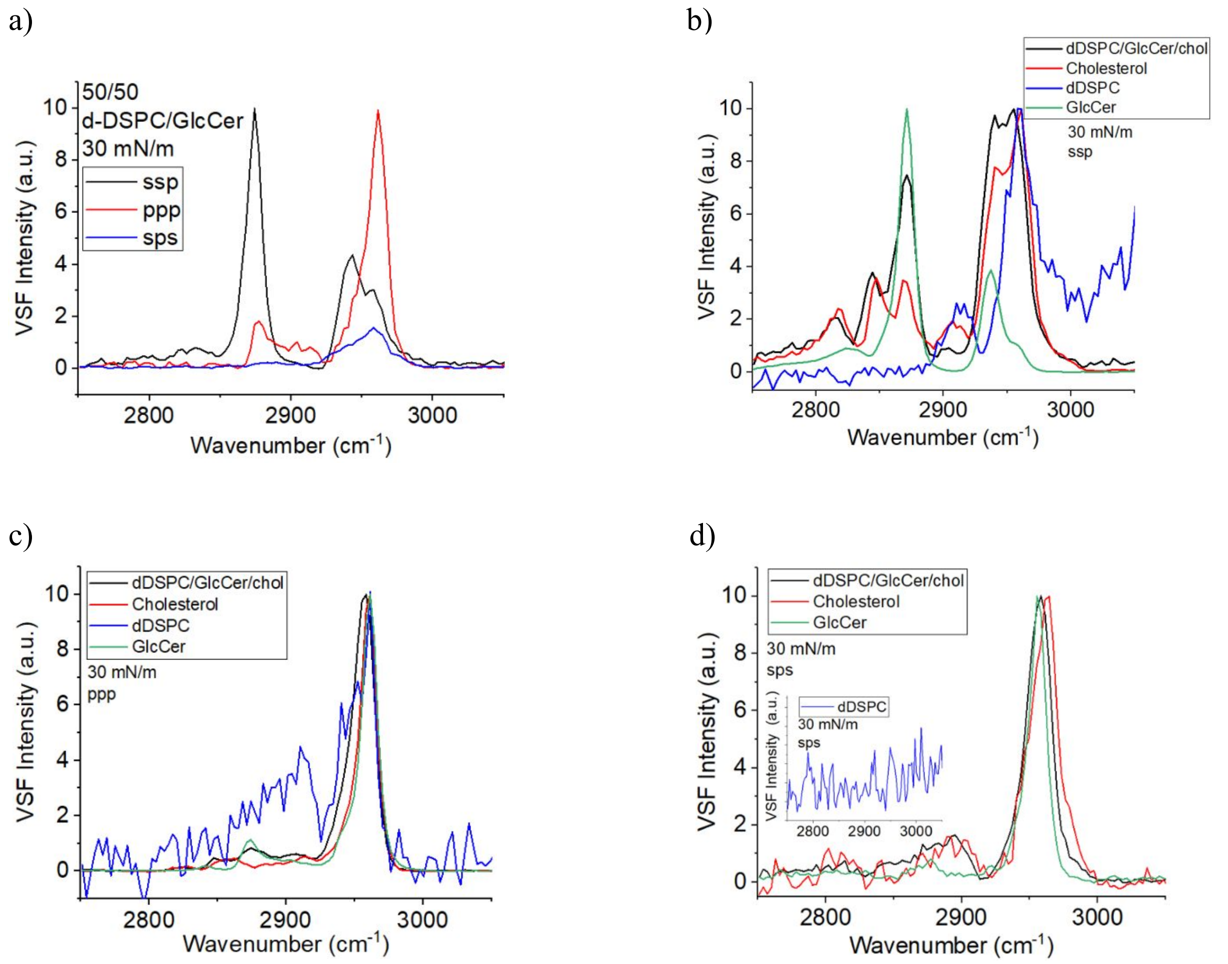

Figure S5: VSF spectra at 30 mN/m showing the a) CH stretching region of the 50/50 mixture of GlcCer and d-DSPC in the ssp, ppp, and sps polarizations, b) the ssp spectrum of the triplet mixture as well as for the individual compounds, c) the ppp spectrum of the triplet mixture and the individual compounds, and d) the sps spectrum of the triplet mixture as well as for the individual compounds. The inset in d) shows the sps spectrum of d-DSPC in the CH stretching region. To visualize peak overlap the intensity of the antisymmetric methyl stretch around 2965 cm^{-1} has been normalized to 10 for all spectra.
